# Breast Cancer Risk Perceptions Among Underserved, Hispanic Women: Implications for Risk-Based Approaches to Screening

**DOI:** 10.1007/s40615-024-01949-7

**Published:** 2024-02-21

**Authors:** Jessica D. Austin, Sarah M. Jenkins, Vera J. Suman, Jhenitza P. Raygoza, Jennifer L. Ridgeway, Aaron Norman, Crystal Gonzalez, Valentina Hernandez, Karthik Ghosh, Bhavika K. Patel, Celine M. Vachon

**Affiliations:** 1 Department of Quantitative Health Sciences, Division of Epidemiology, Mayo Clinic, 13400 E. Shea Blvd, Scottsdale, AZ 85259 USA; 2 Department of Quantitative Health Sciences, Division of Clinical Trials and Biostatistics, Mayo Clinic, Rochester, MN USA; 3 Division of Health Care Delivery Research, Mayo Clinic, Rochester, MN USA; 4 Robert D. and Patricia E. Kern Center for the Science of Health Care Delivery, Mayo Clinic, Rochester, MN USA; 5 Department of Integrated Nutrition Services and Collaborative Research, Mountain Park Health Center, Phoenix, AZ USA; 6 Department of Medicine, Mayo Clinic, Rochester, MN USA; 7 Department of Diagnostic Radiology, Mayo Clinic, Phoenix, AZ USA; 8 Department of Quantitative Health Sciences, Division of Epidemiology, Mayo Clinic, Rochester, MN USA

**Keywords:** Risk perceptions, Breast cancer, Hispanics, Underserved, Screening

## Abstract

**Background:**

Understanding factors that shape breast cancer risk perceptions is essential for implementing risk-based approaches to breast cancer detection and prevention. This study aimed to assess multilevel factors, including prior screening behavior, shaping underserved, Hispanic women’s perceived risk for breast cancer.

**Methods:**

Secondary analysis of survey data from Hispanic women (*N* = 1325, 92% Spanish speaking, 64% < 50) enrolled in a large randomized controlled trial. Analyses were performed in two cohorts to account for the role of age on screening guideline recommendations (< 50 and 50 +). For each cohort, we examined differences in three common measures of perceived risk of breast cancer (percent lifetime, ordinal lifetime, comparative) by participant factors with chi-square or Kruskal–Wallis tests, as appropriate. Multivariate analyses examined the association between mammography history with percent perceived lifetime risk (outcome > 10 vs ≤ 10%).

**Results:**

Overall, 75% reported a lifetime risk between 0 and 10%, 96% rated their ordinal risk as “not high,” and 50% rated their comparative risk as “much lower.” Women < 50 with a family history of breast cancer reported significantly higher levels of perceived risk across all three measures. Among women 50 + , those reporting lower levels of perceived risk were significantly more likely to be Spanish speaking. No significant association was observed between mammography history and percent lifetime risk of breast cancer.

**Conclusion:**

Factors shaping breast cancer risk perceptions differ by age. Prior screening may play less of role in constructing risk perceptions. Research is needed to develop culturally and linguistically appropriate strategies to improve implementation of risk-based screening.

## Introduction

Breast cancer is a leading cause of death among Hispanic women in the USA [1]. Risk-based screening tailored to an individual’s genetic, medical, and socio-behavioral characteristics has the potential to reduce disparities among Hispanic women [2]. Leading organizations, including the US Preventive Services Task Force, the American College of Radiology, and legislative mandates recommend the use of risk-based vs age-based approaches to direct women at high risk for breast cancer to appropriate supplemental screening and risk-reduction strategies [3–6]. Despite availability of evidence-based risk reduction screening strategies, Hispanic women are more likely to be diagnosed at a younger age, with advanced stage disease, and have a lower 5-year survival rate compared to non-Hispanic, White women [7–10]. These disparities have been attributed to complex factors including lack of knowledge of cancer and screening recommendations, lack of access to and use of mammography screening services, fear, and lack of insurance and/or transportation [11–16].

Central to the successful implementation of risk-based screening approaches are women’s understanding of their personal risk for breast cancer. Risk perceptions are complex, contextual evaluations of knowledge that often motivate women to engage in preventive and screening behaviors [17, 18]. Perception of risk for a particular outcome such as breast cancer is often informed by a combination of factors including one’s prior screening experience [19]. However, many women misperceive their risk for breast cancer [20–22], and prior studies show that Hispanic women often perceive their breast cancer risk as low [23–25]. Inaccurate perceptions of personal risk could lead to inappropriate screening schedules and missed opportunities to utilize risk reduction options such as genetic counseling and chemoprevention [4–6, 26].

While the relationship between perceived risk and screening behavior is well documented, few studies have explored factors associated with breast cancer risk perceptions among primarily Spanish-speaking, Hispanic women who are more susceptible to disparities in breast cancer screening and mortality. Thus, the purpose of this study is to understand the associations between patient factors including sociodemographic, clinical, and prior experience, on Hispanic women’s perceived risk for breast cancer.

## Methods

This is a secondary analysis of self-reported baseline survey data from an NIH-funded randomized trial comparing the impact of three breast density educational approaches on behavioral and psychological outcomes [27]. Eligible women in the trial were English or Spanish-speaking Hispanic women, between 40 and 74 years of age, presenting for a screening mammogram at a large Federally Qualified Health Center (FQHC) in Phoenix, AZ. FQHCs are safety net clinics that provide comprehensive healthcare services including primary and preventive care regardless of a patient’s ability to pay [28]. Between October 2016 and October 2019, 1332 Hispanic women were registered and completed the baseline survey. For this analysis, we excluded 7 women who did not respond to any of the self-perceived risk questions or the mammography screening behavior questions (*N* = 1325). This study was approved by the Mayo Clinic Institutional Review Board and all participants provided written informed consent and received financial compensation for their time.

### Data Collection

The baseline survey was administered in-person during a screening appointment and included items on demographic characteristics, family history of breast cancer, mammography screening behavior, health literacy, reproductive history (e.g., parity, age at menarche, menopausal status), and health beliefs including perceived risk of breast cancer. Clinical characteristics from the electronic health record, such as body mass index (BMI), primary family history of breast cancer (first-degree relative with breast cancer), and history of breast biopsy, were also collected at baseline.

#### Perceived Risk of Breast Cancer

The baseline survey included three common measures of perceived risk of breast cancer adapted from prior studies with Hispanics and nationally representative surveys including the Health Information National Trends Survey [29–31]. A numerical risk estimation was obtained by asking participants to estimate their risk of breast cancer in their lifetime using a 0–100% open-ended response (*percent lifetime risk) *[29, 31]. Due to sparse distribution in participant responses (59% of respondents assigned themselves a risk of 0%, 75% self-reported a risk of 0–10%), we dichotomized responses as 0–10% and > 10% to align with current estimates of a Hispanic woman’s lifetime risk of developing breast cancer (9.8%) [32]. Ordinal lifetime risk was assessed by asking participants to rate their lifetime risk on a 5-point ordinal scale [29, 31]. Responses were dichotomized as “very low, moderately low, neither high nor low” and “moderately high or very high.” Participants were also asked to assess their risk compared to women their age with the response options of “much lower,” “about the same,” and “much higher” (*comparative risk)* [30, 31].

#### Mammography Screening History

We defined mammography screening behavior as the number of prior mammograms completed prior to baseline. Participants were asked if they ever had a mammogram before their screening appointment with responses dichotomized as “yes” and “no.” Women responding “yes” were then asked how many total mammograms they think they had in their lifetime. Response options were categorized as “0” (including those with no prior mammogram), “1,” “2–4,” and “5 + .”

## Analysis

Given that guideline recommendations for initiation and frequency of mammography screening are largely driven by age, we divided the study population into two cohorts, namely, those less than 50 years of age (< 50) and those aged 50 or older (50 +) at study entry. For each cohort, chi-square and Kruskal–Wallis tests (for categorical and ordinal variables, respectively) were used to examine whether any of three measures of perceived risk of breast cancer (*percent lifetime, percent ordinal, comparative*) differed by patient social demographic or breast cancer risk factors. Multivariate analyses were performed with logistic regression, using the score method for model selection, to examine the association between mammography history and percent perceived lifetime risk (outcome > 10 vs ≤ 10%) for the overall group and each cohort. The multivariate analysis was limited to perceived lifetime risk due to a small number of women perceiving their ordinal and comparative risk as high. Odds ratios (OR), along with 95% confidence intervals (CI), are reported, and *p*-values less than 0.05 were considered statistically significant. All analyses were performed using SAS version 9.4 (SAS Institute Inc., Cary, NC).

## Results

**Fig. 1:**
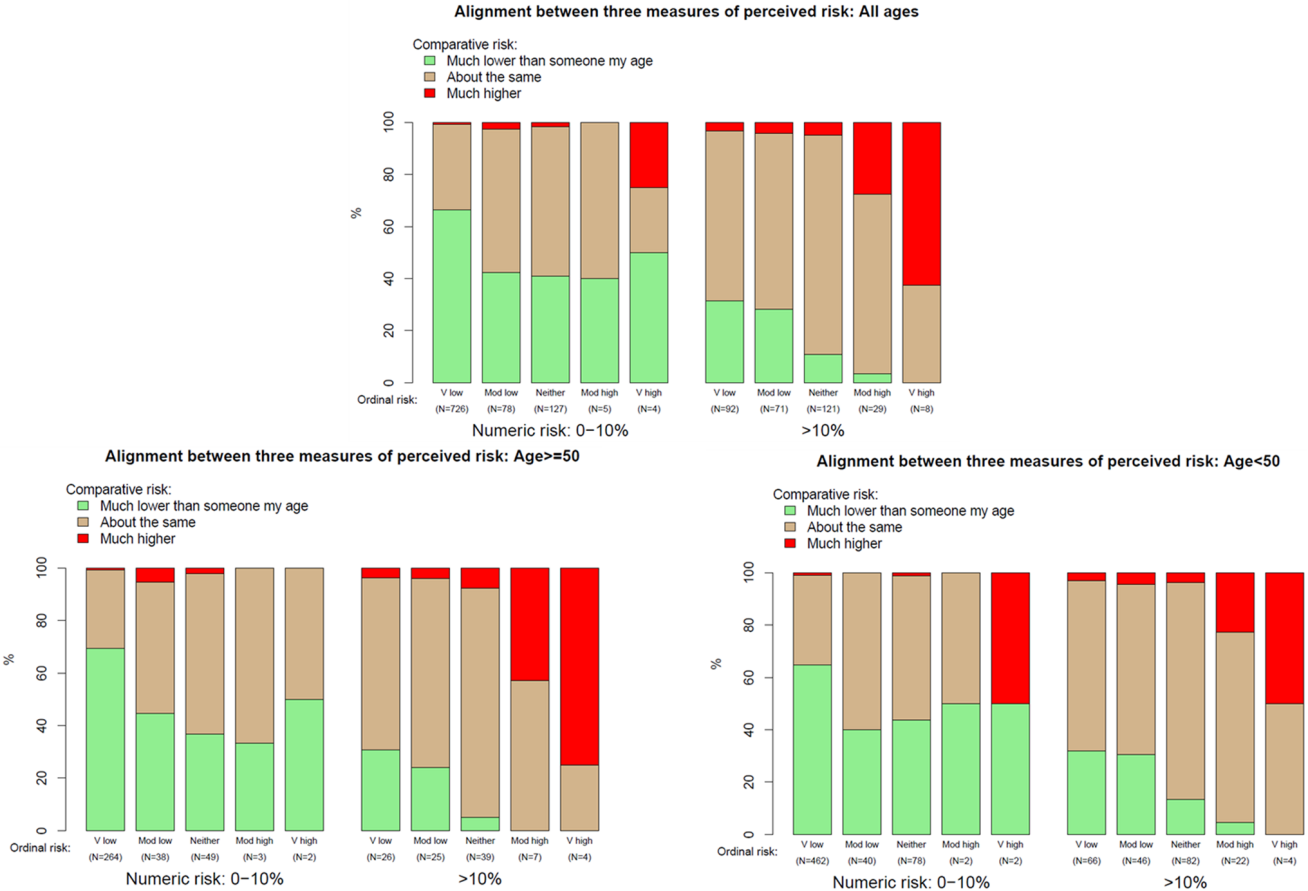
Alignment between three measures of perceived risk overall and by age

Table 1 provides demographic and screening history of the women in our study population. Approximately two-thirds of the women were under the age of 50 (64%) and 69% had less than a high school education. Over 90% of the women completed the survey in Spanish, and 92% never had a breast biopsy or a primary family history of breast cancer. In our sample, perceived risk for breast cancer was low, with 75% considering their percent lifetime risk between 0 and 10%, 96% stating their ordinal risk as “not high,” and 50% describing their comparative risk as “much lower” than peers of similar age. The distributions of lifetime risk, comparative risk, and ordinal lifetime overall and by cohort are presented in Fig. 1. In general, ratings of perceived risk were similarly across all measures. Yet, there are still women who perceived their lifetime risk to be ≤ 10% but consider their ordinal and comparative risk as high.

**Table 1:** Summary of participant characteristics (*N* = 1325)

	*N* (%)
Age	
40–44	480 (36.2%)
45–49	368 (27.8%)
50–54	229 (17.3%)
55 +	248 (18.7%)
Education	
Less than high school	910 (69.0%)
High school or more	408 (31.0%)
Marital status	
Single	424 (32.1%)
Partnered/married	895 (67.9%)
Language at consent	
English	102 (7.7%)
Spanish	1223 (92.3%)
History of breast biopsy	
No	1219 (92.1%)
Yes	105 (7.9%)
Primary family history of breast cancer	
No	1217 (92.1%)
Yes	104 (7.9%)
Parity, mean (SD)	3.37 (1.62)
Menopausal status	
Premenopausal	795 (60.4%)
Postmenopausal	522 (39.6%)
Body mass index (kg/m^2^)	
< 25, normal weight	183 (13.8%)
25–29, overweight	468 (35.4%)
≥ 30, obese	672 (50.8%)
Percent lifetime risk	
0–10%	955 (74.7%)
< 10%	324 (25.3%)
Ordinal lifetime risk	
Not high	1254 (96.3%)
At least moderately high	48 (3.7%)
Comparative risk	
Much lower	658 (49.8%)
About the same	623 (47.2%)
Much higher	39 (3.0%)
Mammography screening history	
0	239 (18.0%)
1	237 (17.9%)
2–4	496 (37.4%)
5 +	353 (26.6%)

Among women under the age of 50 (Table 2), those who considered themselves to have a higher level of risk were significantly more likely to report a primary family history of breast cancer than those reporting lower levels of risk (all three measures, *p* < 0.01). For percent lifetime risk and comparative risk endpoints, but not for ordinal lifetime risk, women who considered themselves to have a higher level of risk were significantly more likely to report English as their preferred language or had a history of breast biopsy than those reporting lower levels of risk (all *p* < 0.05). For the percent lifetime risk endpoint only, women stating their breast cancer risk as > 10% were more likely to have finished high school as compared to those stating risk of 0–10% (*p* < 0.01). Among women aged 50 or older (Table 3), those who reported higher levels of perceived risk across all three measures were also significantly more likely to speak English as their primary language (*p* < 0.05). For the percent lifetime risk and ordinal lifetime risk endpoints, women who considered themselves to have a higher level of risk were significantly more likely to report a primary family history of breast cancer than those reporting lower levels of risk (*p* < 0.01).

**Table 2:** Association of sociodemographic, clinical, and prior experience on perceived risk of breast cancer age < 50

	Percent lifetime risk			Ordinal lifetime risk			Comparative risk			
	0–10% (*N* = 592)	> 10% (*N* = 223)	*p*-value	Not high (*N* = 802)	At least moderately high (*N* = 32)	*p*-value	Much lower (*N* = 417)	About the same (*N* = 405)	Much higher (*N* = 23)	*p*-value
Mammography history										
0	164 (27.7%)	54 (24.2%)	0.72	217 (27.1%)	5 (15.6%)	0.13	113 (27.1%)	109 (26.9%)	2 (8.7%)	0.39
1	132 (22.3%)	56 (25.1%)		181 (22.6%)	11 (34.4%)		91 (21.8%)	97 (24.0%)	7 (30.4%)	
2–4	233 (39.4%)	90 (40.4%)		317 (39.5%)	10 (31.3%)		171 (41.0%)	150 (37.0%)	10 (43.5%)	
5 or more	63 (10.6%)	23 (10.3%)		87 (10.8%)	6 (18.8%)		42 (10.1%)	49 (12.1%)	4 (17.4%)	
Age										
40–44	331 (55.9%)	130 (58.3%)	0.54	459 (57.2%)	15 (46.9%)	0.25	240 (57.6%)	229 (56.5%)	10 (43.5%)	0.41
45–49	261 (44.1%)	93 (41.7%)		343 (42.8%)	17 (53.1%)		177 (42.4%)	176 (43.5%)	13 (56.5%)	
Education										
Less than high school	405 (68.5%)	125 (56.3%)	< 0.01	521 (65.1%)	20 (62.5%)	0.76	275 (66.1%)	259 (64.1%)	11 (47.8%)	0.19
High school or more	186 (31.5%)	97 (43.7%)		279 (34.9%)	12 (37.5%)		141 (33.9%)	145 (35.9%)	12 (52.2%)	
Marital status										
Single	154 (26.1%)	59 (26.7%)	0.85	205 (25.7%)	9 (28.1%)	0.75	104 (25.1%)	106 (26.2%)	6 (26.1%)	0.93
Partnered/married	437 (73.9%)	162 (73.3%)		594 (74.3%)	23 (71.9%)		311 (74.9%)	298 (73.8%)	17 (73.9%)	
Language at consent										
English	24 (4.1%)	18 (8.1%)	0.02	41 (5.1%)	2 (6.3%)	0.78	14 (3.4%)	25 (6.2%)	6 (26.1%)	< 0.01
Spanish	568 (95.9%)	205 (91.9%)		761 (94.9%)	30 (93.8%)		403 (96.6%)	380 (93.8%)	17 (73.9%)	
History of breast biopsy										
No	564 (95.3%)	204 (91.5%)	0.04	753 (93.9%)	30 (93.8%)	0.97	396 (95.0%)	380 (93.8%)	18 (78.3%)	< 0.01
Yes	28 (4.7%)	19 (8.5%)		49 (6.1%)	2 (6.3%)		21 (5.0%)	25 (6.2%)	5 (21.7%)	
Primary family history of breast cancer										
No	570 (96.3%)	193 (86.9%)	< 0.01	758 (94.6%)	23 (71.9%)	< 0.01	401 (96.2%)	374 (92.6%)	16 (69.6%)	< 0.01
Yes	22 (3.7%)	29 (13.1%)		43 (5.4%)	9 (28.1%)		16 (3.8%)	30 (7.4%)	7 (30.4%)	
Parity (mean SD)	3.28 (1.56)	3.22 (1.59)	0.36	3.27 (1.56)	3.19 (1.35)	0.52	3.20 (1.40)	3.34 (1.66)	3.30 (1.92)	0.77
Menopausal status										
Premenopausal	498 (84.7%)	192 (86.1%)	0.62	681 (85.3%)	26 (81.3%)	0.52	354 (85.1%)	342 (85.1%)	20 (87.0%)	0.97
Postmenopausal	90 (15.3%)	31 (13.9%)		117 (14.7%)	6 (18.8%)		62 (14.9%)	60 (14.9%)	3 (13.0%)	
Body mass index										
< 25	79 (13.4%)	37 (16.6%)	0.48	114 (14.3%)	6 (18.8%)	0.60	62 (14.9%)	56 (13.9%)	4 (17.4%)	0.92
25–29	216 (36.6%)	76 (34.1%)		288 (36.0%)	9 (28.1%)		153 (36.8%)	142 (35.1%)	7 (30.4%)	
≥ 30	295 (50.0%)	110 (49.3%)		398 (49.8%)	17 (53.1%)		201 (48.3%)	206 (51.0%)	12 (52.2%)

**Table 3:** Association of sociodemographic, clinical, and prior experience on perceived risk of breast cancer age ≥ 50

	Percent lifetime risk			Ordinal lifetime risk			Comparative risk			
	0–10% (*N* = 363)	> 10% (*N* = 101)	*p*-value	Not high (*N* = 452)	At least moderately high (*N* = 16)	*p*-value	Much lower (*N* = 241)	About the same (*N* = 218)	Much higher (*N* = 16)	*p*-value
Mammography history										
0–1^1^	45 (12.4%)	7 (6.9%)	0.09	52 (11.5%)	1 (6.3%)	0.81	29 (12.0%)	25 (11.5%)	0 (0.0%)	0.28
2–4	129 (35.5%)	30 (29.7%)		157 (34.7%)	6 (37.5%)		84 (34.9%)	70 (32.1%)	9 (56.3%)	
5 or more	189 (52.1%)	64 (63.4%)		243 (53.8%)	9 (56.3%)		128 (53.1%)	123 (56.4%)	7 (43.8%)	
Age										
50–54	166 (45.7%)	52 (51.5%)	0.31	219 (48.5%)	6 (37.5%)	0.39	107 (44.4%)	115 (52.8%)	6 (37.5%)	0.14
≥ 55	197 (54.3%)	49 (48.5%)		233 (51.5%)	10 (62.5%)		134 (55.6%)	103 (47.2%)	10 (62.5%)	
Education										
Less than high school	283 (78.8%)	68 (68.0%)	0.02	344 (77.0%)	10 (62.5%)	0.18	185 (77.1%)	167 (78.0%)	8 (50.0%)	0.04
High school or more	76 (21.2%)	32 (32.0%)		103 (23.0%)	6 (37.5%)		55 (22.9%)	47 (22.0%)	8 (50.0%)	
Marital status										
Single	162 (44.9%)	41 (40.6%)	0.44	194 (43.2%)	9 (56.3%)	0.30	117 (48.5%)	81 (37.7%)	7 (43.8%)	0.06
Partnered/married	199 (55.1%)	60 (59.4%)		255 (56.8%)	7 (43.8%)		124 (51.5%)	134 (62.3%)	9 (56.3%)	
Language at consent										
English	38 (10.5%)	18 (17.8%)	0.04	51 (11.3%)	6 (37.5%)	< 0.01	27 (11.2%)	24 (11.0%)	6 (37.5%)	< 0.01
Spanish	325 (89.5%)	83 (82.2%)		401 (88.7%)	10 (62.5%)		214 (88.8%)	194 (89.0%)	10 (62.5%)	
History of breast biopsy										
No	320 (88.4%)	91 (90.1%)	0.63	398 (88.2%)	16 (100.0%)	0.15	217 (90.0%)	187 (86.2%)	16 (100.0%)	0.15
Yes	42 (11.6%)	10 (9.9%)		53 (11.8%)	0 (0.0%)		24 (10.0%)	30 (13.8%)	0 (0.0%)	
Primary family history of breast cancer										
No	333 (92.0%)	79 (79.8%)	< 0.01	405 (90.2%)	10 (62.5%)	< 0.01	218 (91.2%)	191 (88.0%)	12 (75.0%)	0.10
Yes	29 (8.0%)	20 (20.2%)		44 (9.8%)	6 (37.5%)		21 (8.8%)	26 (12.0%)	4 (25.0%)	
Parity (mean SD)	3.61 (1.78)	3.39 (1.57)	0.25	3.55 (1.68)	3.50 (2.07)	0.92	3.67 (1.74)	3.46 (1.71)	3.25 (1.91)	0.27
Menopausal status										
Premenopausal	57 (15.9%)	17 (16.8%)	0.82	73 (16.3%)	2 (12.5%)	0.69	34 (14.3%)	39 (18.0%)	2 (12.5%)	0.52
Postmenopausal	302 (84.1%)	84 (83.2%)		375 (83.7%)	14 (87.5%)		204 (85.7%)	178 (82.0%)	14 (87.5%)	
Body mass index										
< 25	46 (12.7%)	13 (12.9%)	0.41	59 (13.1%)	0 (0.0%)	0.12	32 (13.3%)	28 (12.8%)	1 (6.3%)	0.09
25–29	133 (36.6%)	30 (29.7%)		160 (35.4%)	4 (25.0%)		96 (39.8%)	67 (30.7%)	3 (18.8%)	
≥ 30	184 (50.7%)	58 (57.4%)		233 (51.5%)	12 (75.0%)		113 (46.9%)	123 (56.4%)	12 (75.0%)	

^1^Combining 0–1 prior mammograms together due to sparsity

Next, we examined associations with the likelihood of a woman perceiving her risk of developing breast cancer as > 10% (Table 4). No statistically significant association was found between number of prior mammograms and the likelihood a woman perceives her risk of developing breast cancer as > 10% after adjusting for age, education, primary language, and a primary family history of breast cancer. Examining this question in the cohort of women < 50 years of age as well as the cohort of women ≥ 50 years of age revealed no statistically significant association between number of prior mammograms and the likelihood a woman perceives her risk of developing breast cancer as > 10% after adjusting for education level attained and primary family history of breast cancer.

**Table 4:** Results of logistic regression modeling the odds of perceived risk (> 10 vs 0–10%) based on mammography screening history behavior by age

		Univariate modeling results		Multivariate modeling results	
Group	Mammography history (# mammograms)	OR (95% CI)	*p*-value	OR (95% CI)	Adjusted *p*-value
All ages	0	Reference	0.82	Reference	0.42^1^
	1	1.22 (0.80 to 1.85)		1.24 (0.80 to 1.92)	
	2–4	1.07 (0.74 to 1.54)		1.13 (0.76 to 1.70)	
	5 +	1.11 (0.75 to 1.64)		1.47 (0.90 to 2.40)	
Age < 50	0	Reference	0.72	Reference	0.82^2^
	1	1.29 (0.83 to 2.00)		1.20 (0.76 to 1.87)	
	2–4	1.17 (0.79 to 1.74)		1.02 (0.68 to 1.53)	
	5 +	1.11 (0.63 to 1.96)		0.95 (0.53 to 1.71)	
Age ≥ 50	0–1^3^	Reference	0.10	Reference	0.21^2^
	2–4	1.50 (0.61 to 3.64)		1.39 (0.56 to 3.41)	
	5 +	2.18 (0.94 to 5.07)		1.91 (0.81 to 4.49)	

^1^Adjusting for age group (40–44, 45–49, 50–54, or 55 +), education (high school or more vs less than high school), language (Spanish vs English), and family history of breast cancer (yes vs no)

^2^Adjusting for education (high school or more vs less than high school), and family history of breast cancer (yes vs no)

^3^Combining 0–1 prior mammograms together due to sparsity

## Discussion

In this study, we aimed to identify factors associated with perceived risk for breast cancer in a largely understudied population and to assess the relationship between mammography screening history and perceived risk. Overall, Hispanic women’s perceptions of their breast cancer risk was low, and we observed no significant associations between mammography screening history and percent perceived lifetime risk of breast cancer. Our findings are consistent with the limited number of studies inclusive of Hispanic women [23, 25, 30, 33], and may reflect issues in appropriate and acceptable cancer risk and prevention messaging, greater presence of competing risks, and salience of risk perceptions [30, 34].

Lived experiences are believed to play a key role in constructing risk perceptions. For instance, having consecutive normal mammogram results may be interpreted by the patient as a clean bill of health and evidence of low cancer risk. While getting regular mammograms lowers one’s risk of dying from breast cancer, it does not eliminate one’s risk of getting breast cancer. It is possible that women in our sample misperceived the act of engaging in preventive care, such as obtaining a mammogram, would reduce their risk of developing breast cancer. However, prior screening history was not significantly associated with any of the three measures of perceived risk in our sample. These findings support that the relationship between risk perceptions and screening behavior may be less salient among Hispanic women, compared to non-Hispanic White and Black women, and that other factors such as education and prior family history may play a larger role in shaping risk perceptions [30, 34].

Moreover, 92% of women in our sample spoke Spanish as their primary language. It is well documented that Spanish-speaking, Hispanic women experience numerous barriers to care that may overwhelm the motivational impact of perceiving one to be at risk for breast cancer. However, it is important to note that all women in our study were recruited at the time of a mammography screening appointment suggesting that they were able to overcome barriers to access and use. To this end, the low levels of perceived risk we observed may reflect low levels of knowledge, awareness, or communication around a woman’s personal risk for breast cancer. Improving knowledge and awareness of breast cancer risk factors is central to risk communication and should be considered alongside beliefs and values around risk factors. The term “risk” may also hold different meaning for Spanish-speaking women compared to experts and other groups [35]. More research is needed to understand Hispanic women’s knowledge of risk factors and how these factors shape risk perceptions. These efforts should also be considered alongside complex social and structural influences (i.e., access to care, cost, transportation, literacy) that have shown to hinder uptake of breast cancer preventive care and drive existing disparities in breast cancer outcomes [23, 36].

Prior studies among Hispanic women found that family history was not sufficient to increase breast cancer risk perceptions [30, 37]. However, we found that primary family history was associated with higher levels of perceived risk across all three measures among Hispanic women under the age of 50 and associated with a > 10% lifetime risk and at least moderately high ordinal risk among Hispanic women aged 50 and older. Yet, breast cancer resulting from familial predisposition is thought to account for only 15 to 25% of all diagnosed cases [38, 39]. Overreliance on family history of breast cancer to determine one’s own breast cancer risk may skew not only breast cancer risk perception, but may also affect rates of repeat mammography screening [40]. Additionally, we observed differences in at least two of the measures of perceived risk by language among women of both cohorts. This could reflect how women think about the term “risk.” Risk and the beliefs about the causes, curability, and risk factors of breast cancer differ for lay women compared to health care experts [41], and this difference may be more pronounced in non-English speaking populations. Thus, more research is needed to improve our understanding of how Spanish-speaking, Hispanic women understand and appraise their risk to inform communication strategies that are essential for enhancing implementation of risk-based screening approaches.

## Strengths and Limitations

The cross-sectional nature of our study design limits causal interpretation since all measures were collected at the same time. All participants were also recruited from a single FQHC clinic during the time of a mammogram appointment limiting generalizability and possibly biasing our findings. Given that all participants were receiving preventive care, it is possible that they believed receipt of preventive care lowers one’s breast cancer risk. It is also possible that low levels of risk observed in our population were appropriate. A study by our team found that 6.8% of women in our sample had an estimated Gail Model risk > 10%, but nearly 50% of women with an estimated risk > 10% reported their perceived lifetime risk to be less than 10% [42]. However, the homogenous nature of our sample accessing screening services provides critical insights into how similar populations think about their breast cancer risk and additional factors potentially contributing to low levels of risk (poor provider communication, lack of knowledge). As previously mentioned, we observed inconsistencies in how women in our sample responded to three common measures of perceived risk, suggesting that these measures may lack cultural resonance. These inconsistencies, combined with the decision to dichotomize percent lifetime risk, may have limited our ability to understand factors associated with one’s perceived risk for breast cancer.

## Conclusion

Improving risk communication and perceptions, particularly among those at high risk, is crucial for implementing risk-based screening and risk reduction strategies. Our findings suggest that the perceived risk for breast cancer, as conceptualized in common measures by prevention science and health behavior theory, may lack cultural resonance or play a less important role in screening behavior. Future studies are needed to understand how underserved, Spanish-speaking, Hispanic women think about and formulate their breast cancer risk to inform the development of strategies to improve risk communication in a manner that is culturally and linguistically appropriate. Potential strategies may include the use of community health workers or Promotoras to deliver education around breast cancer risk and risk-reduction strategies and efforts to improve provider knowledge, assessment, and communication skills around breast cancer risk. However, an unintended consequence of informing women about their risk is an increase in worry or concern [43, 44] and the inability to provide high-risk women receiving care in FQHC with guideline recommended supplemental screening or chemoprevention, which may cause more distress and exacerbate disparities. These factors should be considered as we continue to shift from age-based to more risk-based approaches to breast cancer screening and treatment.

## Data Availability

The data underlying this article cannot be publicly shared due to existing consent and Institutional Review Board constraints. Additional summary level data without individual data may be shared upon request and permission from community partners who supported this project.

## References

[1] American Cancer Society. Cancer facts & figures for Hispanic/Latino people 2021–2023. Atlanta: American Cancer Society; 2021.

[2] Schwartz C, et al. Association of population screening for breast cancer risk with use of mammography among women in medically underserved racial and ethnic minority groups. JAMA Netw Open. 2021;4(9):e2123751–e2123751. 34505886 10.1001/jamanetworkopen.2021.23751 PMC8433603

[3] Dense Breast Info. Legistlation and regulations - what is required?: Densebreast-info.org. Available from: http://densebreast-info.org/legislation.aspx#. Accessed May 2023.

[4] Force UPST. Medication use to reduce risk of breast cancer: US Preventive Services Task Force recommendation statement. JAMA. 2019;322(9):857–67. 31479144 10.1001/jama.2019.11885

[5] Monticciolo DL, et al. Breast cancer screening recommendations inclusive of all women at average risk: update from the ACR and society of breast imaging. J Am Coll Radiol. 2021;18(9):1280–8. 34154984 10.1016/j.jacr.2021.04.021

[6] Nelson HD, et al. Risk assessment, genetic counseling, and genetic testing for BRCA-related cancer in women: updated evidence report and systematic review for the US Preventive Services Task Force. JAMA. 2019;322(7):666–85. 31429902 10.1001/jama.2019.8430

[7] Society AC. Cancer facts & figures for Hispanic/Latino people 2021–2023. Atlanta: American Cancer Society, Inc; 2021.

[8] Iqbal J, et al. Differences in breast cancer stage at diagnosis and cancer-specific survival by race and ethnicity in the United States. JAMA. 2015;313(2):165–73. 25585328 10.1001/jama.2014.17322

[9] Nahleh Z, et al. Disparities in breast cancer: a multi-institutional comparative analysis focusing on American Hispanics. Cancer Med. 2018;7(6):2710–7. 29733543 10.1002/cam4.1509 PMC6010853

[10] Boone SD, et al. The joint contribution of tumor phenotype and education to breast cancer survival disparity between Hispanic and non-Hispanic white women. Cancer Causes Control. 2014;25(3):273–82. 24337810 10.1007/s10552-013-0329-3

[11] Gangnon RE, et al. The contribution of mammography screening to breast cancer incidence trends in the United States: an updated age-period-cohort model. Cancer Epidemiol Biomarkers Prev. 2015;24(6):905–12. 25787716 10.1158/1055-9965.EPI-14-1286 PMC4489135

[12] Austin LT, et al. Breast and cervical cancer screening in Hispanic women: a literature review using the health belief model. Womens Health Issues. 2002;12(3):122–8. 12015184 10.1016/s1049-3867(02)00132-9

[13] Wells KJ, Roetzheim RG. Health disparities in receipt of screening mammography in Latinas: a critical review of recent literature. Cancer Control. 2007;14(4):369–79. 17914337 10.1177/107327480701400407

[14] Shelton RC, et al. Sociocultural determinants of breast and cervical cancer screening adherence: an examination of variation among immigrant Latinas by country of origin. J Health Care Poor Underserved. 2012;23(4):1768–92. 23698689 10.1353/hpu.2012.0191

[15] Nuño T, et al. Breast and cervical cancer screening utilization among Hispanic women living near the United States-Mexico border. J Womens Health (Larchmt). 2011;20(5):685–93. 21428792 10.1089/jwh.2010.2205

[16] Rauscher GH, et al. Disparities in screening mammography services by race/ethnicity and health insurance. J Womens Health (Larchmt). 2012;21(2):154–60. 21942866 10.1089/jwh.2010.2415 PMC3270049

[17] Janz NK, Becker MH. The Health Belief Model: a decade later. Health Educ Q. 1984;11(1):1–47. 6392204 10.1177/109019818401100101

[18] Vernon SW. Risk perception and risk communication for cancer screening behaviors: a review. J Natl Cancer Inst Monogr. 1999;25:101–19. 10.1093/oxfordjournals.jncimonographs.a024184 10854465

[19] Weinstein ND. Unrealistic optimism about susceptibility to health problems: conclusions from a community-wide sample. J Behav Med. 1987;10(5):481–500. 3430590 10.1007/BF00846146

[20] Andersen MR, et al. Breast cancer worry and mammography use by women with and without a family history in a population-based sample. Cancer Epidemiol Biomarkers Prev. 2003;12(4):314–20. 12692105

[21] Black WC, Nease RF Jr, Tosteson AN. Perceptions of breast cancer risk and screening effectiveness in women younger than 50 years of age. J Natl Cancer Inst. 1995;87(10):720–31. 7563149 10.1093/jnci/87.10.720

[22] Gross CP, et al. The relation between projected breast cancer risk, perceived cancer risk, and mammography use Results from the National Health Interview Survey. J Gen Intern Med. 2006;21(2):158–64. 16390511 10.1111/j.1525-1497.2005.00312.x PMC1484644

[23] Graves KD, et al. Perceived risk of breast cancer among Latinas attending community clinics: risk comprehension and relationship with mammography adherence. Cancer Causes Control. 2008;19(10):1373–82. 18704716 10.1007/s10552-008-9209-7 PMC4309545

[24] Fehniger J, et al. Perceived versus objective breast cancer risk in diverse women. J Womens Health (Larchmt). 2014;23(5):420–7. 24372085 10.1089/jwh.2013.4516 PMC4011422

[25] Orom H, et al. Perceived cancer risk: why is it lower among nonwhites than whites? Cancer Epidemiol Biomarkers Prev. 2010;19(3):746–54. 20160278 10.1158/1055-9965.EPI-09-1085 PMC2836595

[26] Monticciolo DL, et al. Breast cancer screening in women at higher-than-average risk: recommendations from the ACR. J Am Coll Radiol. 2018;15(3 Pt A):408–14. 29371086 10.1016/j.jacr.2017.11.034

[27] Patel BK, et al. Behavioral and psychological impact of returning breast density results to Latinas: study protocol for a randomized clinical trial. Trials. 2019;20(1):744. 31852492 10.1186/s13063-019-3939-6 PMC6921571

[28] Allen CL, et al. Opportunities for improving cancer prevention at federally qualified health centers. J Cancer Educ. 2014;29(1):30–7. 23996232 10.1007/s13187-013-0535-4 PMC3920058

[29] Gurmankin Levy A, et al. Measuring perceptions of breast cancer risk. Cancer Epidemiol Biomarkers Prev. 2006;15(10):1893–8. 17035396 10.1158/1055-9965.EPI-05-0482

[30] Orom H, et al. Perceived risk for breast cancer and its relationship to mammography in Blacks, Hispanics, and Whites. J Behav Med. 2013;36(5):466–76. 22772713 10.1007/s10865-012-9443-z PMC3565065

[31] Finney Rutten LJ, et al. Data resource profile: the National Cancer Institute’s Health Information National Trends Survey (HINTS). Int J Epidemiol. 2019;49(1):17–17j. 10.1093/ije/dyz083 PMC7124481 31038687

[32] Power EJ, Chin ML, Haq MM. Breast cancer incidence and risk reduction in the Hispanic population. Cureus. 2018;10(2):e2235. 29713580 10.7759/cureus.2235 PMC5919763

[33] Ramirez AG, et al. Hispanic women’s breast and cervical cancer knowledge, attitudes, and screening behaviors. Am J Health Promot. 2000;14(5):292–300. 11009855 10.4278/0890-1171-14.5.292

[34] Shelton RC, et al. An investigation into the social context of low-income, urban Black and Latina women: implications for adherence to recommended health behaviors. Health Educ Behav. 2011;38(5):471–81. 21856885 10.1177/1090198110382502 PMC3331787

[35] Slovic P. Perception of Risk. Science. 1987;236(4799):280–5. 3563507 10.1126/science.3563507

[36] Coughlin SS. Social determinants of breast cancer risk, stage, and survival. Breast Cancer Res Treat. 2019;177(3):537–48. 31270761 10.1007/s10549-019-05340-7

[37] Ponce NA, et al. Disparities in cancer screening in individuals with a family history of breast or colorectal cancer. Cancer. 2012;118(6):1656–63. 22009719 10.1002/cncr.26480 PMC3262934

[38] Weitzel JN, et al. Prevalence and type of BRCA mutations in Hispanics undergoing genetic cancer risk assessment in the southwestern United States: a report from the Clinical Cancer Genetics Community Research Network. J Clin Oncol. 2013;31(2):210–6. 23233716 10.1200/JCO.2011.41.0027 PMC3532393

[39] Cruz-Correa M, et al. Hereditary cancer syndromes in Latino populations: genetic characterization and surveillance guidelines. Hered Cancer Clin Pract. 2017;15(1):3. 28127413 10.1186/s13053-017-0063-z PMC5251307

[40] Haber G, Ahmed NU, Pekovic V. Family history of cancer and its association with breast cancer risk perception and repeat mammography. Am J Public Health. 2012;102(12):2322–9. 23078489 10.2105/AJPH.2012.300786 PMC3519312

[41] Silverman E, et al. Women’s views on breast cancer risk and screening mammography: a qualitative interview study. Med Decis Making. 2001;21(3):231–40. 11386630 10.1177/0272989X0102100308

[42] Karthik Ghosh SMJ, Ridgeway JL, Austin JD, Borah BJ, Patel B, Rhodes DJ, et al. Evaluating the impact of breast density notification on anxiety, breast cancer worry, and self-perceived risk among Latinas at a federally qualified health center. Abstract. 2022 San Antonio Breast Cancer Symposium. December 6-10, 2022

[43] Consedine NS, et al. Fear, anxiety, worry, and breast cancer screening behavior: a critical review. Cancer Epidemiol Biomarkers Prev. 2004;13(4):501–10. 15066912

[44] Skinner CS, et al. Perceived and actual breast cancer risk: optimistic and pessimistic biases. J Health Psychol. 1998;3(2):181–93. 22021358 10.1177/135910539800300203

